# Secondary renal amyloidosis in a patient of pulmonary tuberculosis and common variable immunodeficiency

**Published:** 2015-06-21

**Authors:** Manish R Balwani, Vivek B Kute, Pankaj R Shah, Pawan Wakhare, Hargovind L Trivedi

**Affiliations:** ^1^Department of Nephrology and Clinical Transplantation and Institute of Kidney Diseases and Research Center, Dr. HL Trivedi Institute of Transplantation Sciences (IKDRC-ITS), Ahmedabad, India

**Keywords:** Immunodeficiency, Secondary amyloidosis, Tuberculosis, Proteinuria

## Abstract

Common variable immunodeficiency (CVID) usually manifests in the second or third decade of life with recurrent bacterial infections and hypoglobulinemia. Secondary renal amyloidosis with history of pulmonary tuberculosis is rare in CVID, although T cell dysfunction has been reported in few CVID patients. A 40-year-old male was admitted to our hospital with a 3-month history of recurrent respiratory infections and persistent pitting pedal edema. His past history revealed 3 to 5 episodes of recurrent respiratory tract infections and diarrhoea each year since last 20 years. He had been successfully treated for sputum positive pulmonary tuberculosis 8 years back. Laboratory studies disclosed high erythrocyte sedimentation rate (ESR), hypoalbuminemia and nephrotic range proteinuria. Serum immunoglobulin levels were low. CD4/CD8 ratio and CD3 level was normal. C3 and C4 complement levels were normal. Biopsy revealed amyloid A (AA) positive secondary renal amyloidosis. Glomeruli showed variable widening of mesangial regions with deposition of periodic schiff stain (PAS) pale positive of pink matrix showing apple green birefringence on Congo-red staining. Immunohistochemistry was AA stain positive. Immunofluorescence microscopy revealed no staining with anti-human IgG, IgM, IgA, C3, C1q, kappa and lambda light chains antisera. Patient was treated symptomatically for respiratory tract infection and was discharged with low dose angiotensin receptor blocker. An old treated tuberculosis and chronic inflammation due to recurrent respiratory tract infections were thought to be responsible for AA amyloidosis. Thus pulmonary tuberculosis should be considered in differential diagnosis of secondary causes of AA renal amyloidosis in patients of CVID especially in endemic settings.

**Implication for health policy/practice/research/medical education:**

Common variable immunodeficiency (CVID) usually manifests in the second or third decade of life with recurrent bacterial infections and hypoglobulinemia. Secondary renal amyloidosis with pulmonary tuberculosis is rare in CVID, although T cell dysfunction has been reported in few CVID patients. This case highlights that pulmonary tuberculosis should be considered in differential diagnosis of secondary causes of AA renal amyloidosis in a patient of CVID especially in endemic settings.

## Introduction

Common variable immunodeficiency (CVID) usually manifests in the second or third decade of life with recurrent bacterial infections and hypoglobulinemia (1,2). Chronic diarrhoea is reported in up to 30% of such patients. In less than half of cases, a microbial agent can be identified and is likely to be *Giardia lamblia* or can be due to bacterial overgrowth in the small bowel (3). Recurrent bacterial infections during the course of CVID are known to occur; however, the development of tuberculosis and secondary amyloidosis is uncommon. In our case, we report a case of amyloid A (AA) amyloidosis in CVID probably secondary to tuberculosis and repeated respiratory infections.

## Case presentation

**Figure 1:**
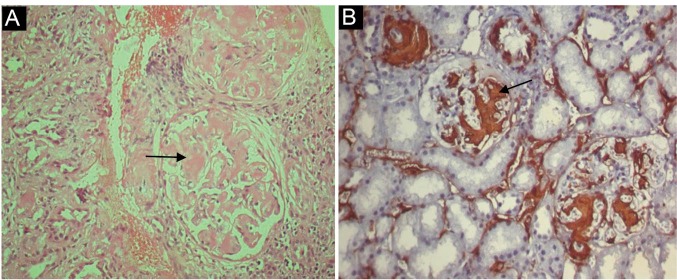
Histologic findings in renal biopsy. A) Glomeruli showed variable widening of mesangial regions with deposition of acellular pale pink matrix (arrow). B) Immunohistochemistry shows AA stain positive (arrow). Original magnification X 200 in A, X 200 in B.

A 40-year-old male was admitted to our hospital with a 3-month history of recurrent respiratory infections and persistent pitting pedal edema. His past history revealed 3 to 5 episodes of recurrent respiratory tract infections and diarrhoea each year since last 20 years. He had been successfully treated for sputum positive pulmonary tuberculosis 8 years back for which he was prescribed isoniazid (300 mg/day), rifampicin (600 mg/day), ethambutol (1.5 g/day) and pyrazinamide (2 g/day) for 3 months, followed by isoniazid and rifampicin for 6 more months. Sputum smear examination for acid fast bacillus (AFB) was negative at 3 and 9 months of treatment. Physical examination revealed a malnourished afebrile male with blood pressure 100/60 mm Hg; and a heart rate of 96/min with pitting pedal edema in both lower limbs. Laboratory studies disclosed; hemoglobin level of 12 g/dl, white blood cells (WBC); 17270/μl, platelets; 641000/μl, erythrocyte sedimentation rate (ESR); 110 mm/h, C-reactive protein (CRP); 32 mg/dl (normal 0-5), total protein; 2.4 g/dl; and serum albumin of 1.4 g/dl. Liver function tests were normal. Urinalysis revealed 3+ proteinuria. Twenty four-hour urine protein was 3.4 g/day. Serum immunoglobulins were as follows: IgG; 1.28 g/l (normal 7-16), IgM ; <0.4 g/l (normal 0.4-2.3); IgA ; <0.6 g/l (normal 0.7-4). CD4/CD8 ratio and CD3 level was normal. C3 and C4 complement levels were normal. The abdominal and pelvic ultrasonography (USG) revealed that the liver was normal in size with a homogenous parenchyma. The spleen was also of normal size, and the parenchymal echotexture was homogenous. Both kidneys were enlarged (right kidney; 120 mm and left kidney; 130 mm) with normal corticomedullary differentiation. Chest x-ray and Electrocardiography (ECG) were normal. Cultures of blood and urine were negative. Serologies for HIV, hepatitis B virus (HBV), and hepatitis C virus (HCV) were negative. Thoracic computed tomography (CT) scan revealed fibrotic scars with bronchiectatic changes in both upper lobes of lungs, suggestive of old healed tuberculosis. Since the patient had recurrent infections, hypogammaglobulinemia, nephrotic level proteinuria, and enlarged kidneys in the renal USG we conducted a renal biopsy. Biopsy revealed, AA positive secondary renal amyloidosis. Glomeruli showed variable widening of mesangial regions with deposition of periodic schiff stain (PAS) pale positive pink matrix showing apple green birefringence on Congo-red staining under polarizer. Immunohistochemistry was AA stain positive (Figure 1). Immunofluorescence microscopy revealed no staining with anti-human IgG, IgM, IgA, C3, C1q, kappa and lambda light chains antisera. Patient was treated symptomatically for respiratory tract infection and was discharged with low dose angiotensin receptor blocker.

## Discussion

Pulmonary tuberculosis in patients with CVID has been rarely reported in the literature (4). Whether this concurrent tuberculosis is just due to a defect in T-cell function is not clear as tuberculosis is a common disease in India. T-cell dysfunction can be seen in up to half of CVID cases (3,5). Although recurrent infections are common in CVID (3-5), no other reported case of secondary renal amyloidosis accompanying CVID, could be traced. Renal amyloidosis secondary to pulmonary tuberculosis is fairly common (6,7). However, old fibrotic changes with bronchiectasis in upper lobes of lung suggests that the patient had a long period of chronic persistent inflammation which may have been a predisposing factor for secondary renal amyloidosis. In secondary renal amyloidosis, the build-up consists of serum amyloid A (SAA) which is an acute phase protein synthesised in the liver, and this condition is called AA amyloidosis. In this case, the SAA that is in the form of a β-folding and is produced in connection with chronic and recurrent infections accumulates extracellularly and leads to damage (8,9). Our patient had nephrotic level proteinuria due to kidney involvement, hypoalbuminemia and pedal edema. Although urine sediments of the patients with CVID and AA amyloidosis may contain a greater number of erythrocytes compared to the AL type of amyloidosis, the repeated urine tests in our patient did not reveal any erythrocytes. Also, since patients with the CVID disease generally have asymptomatic proteinuria, it is possible that the connection between the proteinuria and amyloidosis is overlooked during the course of the underlying disease, which may be an important cause leading to a belated diagnosis. In case of AA amyloidosis that is seen secondary to the CVID, the inflammation continues for an average of around 8 to 14 years before the amyloidosis is diagnosed. Thus, repeated infectious episodes that had occurred since the childhood and the inefficient treatment of these infections lead to the development of AA amyloidosis (10). Our patient’s history revealed that he had frequent episodes of respiratory infections and diarrhoea since last 20 years and had to be hospitalized due to pneumonia at least once in a year. In the past the resolution of symptoms with oral metronidazole in each attack of diarrhoea, suggests that either bacterial overgrowth or giardiasis were responsible for the diarrhoea. It is also known that he had pulmonary tuberculosis 8 years ago for which he was treated successfully. Thus he has completed the necessary time for the development of renal amyloidosis. It is unusual to see tuberculosis with CVID. However, the coincidence of this occurring, in a country endemic for tuberculosis, is not surprising. Secondary amyloidosis in this case was most probably due to the additive effects of tuberculosis and recurrent bacterial infections during the course of CVID. Treatment with IVIG may decrease the recurrent infectious episodes and thus the development of systemic amyloidosis.

## Conclusion

An old treated tuberculosis and chronic inflammation due to recurrent respiratory tract infections were thought to be responsible for the AA amyloidosis. This case highlights that pulmonary tuberculosis should be considered in differential diagnosis of secondary causes of AA renal amyloidosis in a patient of CVID especially in endemic settings.

## Authors’ contribution

All authors wrote the paper equally.

## Conflicts of interest

The authors declared no competing interests.

## Ethical considerations

Ethical issues (including plagiarism, data fabrication, double publication) have been completely observed by the authors.

## Funding/Support

None.
